# Comparison of four methods to measure haemoglobin concentrations in whole blood donors (COMPARE): A diagnostic accuracy study

**DOI:** 10.1111/tme.12750

**Published:** 2020-12-20

**Authors:** Steven Bell, Michael Sweeting, Anna Ramond, Ryan Chung, Stephen Kaptoge, Matthew Walker, Thomas Bolton, Jennifer Sambrook, Carmel Moore, Amy McMahon, Sarah Fahle, Donna Cullen, Susan Mehenny, Angela M. Wood, Jane Armitage, Willem H. Ouwehand, Gail Miflin, David J. Roberts, John Danesh, Emanuele Di Angelantonio, COMPARE Study Group

**Affiliations:** ^1^ Department of Public Health and Primary Care University of Cambridge Cambridge UK; ^2^ Department of Health Sciences University of Leicester Leicester UK; ^3^ NHS Blood and Transplant London, Oxford, Cambridge UK; ^4^ Department of Haematology University of Cambridge Cambridge UK; ^5^ Anglia Ruskin University Chelmsford UK; ^6^ Nuffield Department of Population Health University of Oxford Oxford UK; ^7^ Radcliffe Department of Medicine and BRC Biomedical Centre – Haematology Theme, John Radcliffe Hospital University of Oxford Oxford UK

**Keywords:** gravimetry, haemoglobin screening, HemoCue, inappropriate bleeding, inappropriate deferral, non‐invasive haemoglobin measurement, whole blood donor

## Abstract

**Objective:**

To compare four haemoglobin measurement methods in whole blood donors.

**Background:**

To safeguard donors, blood services measure haemoglobin concentration in advance of each donation. NHS Blood and Transplant's (NHSBT) customary method have been capillary gravimetry (copper sulphate), followed by venous spectrophotometry (HemoCue) for donors failing gravimetry. However, NHSBT's customary method results in 10% of donors being inappropriately bled (ie, with haemoglobin values below the regulatory threshold).

**Methods:**

We compared the following four methods in 21 840 blood donors (aged ≥18 years) recruited from 10 NHSBT centres in England, with the Sysmex XN‐2000 haematology analyser, the reference standard: (1) NHSBT's customary method; (2) “post donation” approach, that is, estimating current haemoglobin concentration from that measured by a haematology analyser at a donor's most recent prior donation; (3) “portable haemoglobinometry” (using capillary HemoCue); (4) non‐invasive spectrometry (using MBR Haemospect or Orsense NMB200). We assessed sensitivity; specificity; proportion who would have been inappropriately bled, or rejected from donation (“deferred”) incorrectly; and test preference.

**Results:**

Compared with the reference standard, the methods ranged in test sensitivity from 17.0% (MBR Haemospect) to 79.0% (portable haemoglobinometry) in men, and from 19.0% (MBR Haemospect) to 82.8% (portable haemoglobinometry) in women. For specificity, the methods ranged from 87.2% (MBR Haemospect) to 99.9% (NHSBT's customary method) in men, and from 74.1% (Orsense NMB200) to 99.8% (NHSBT's customary method) in women. The proportion of donors who would have been inappropriately bled ranged from 2.2% in men for portable haemoglobinometry to 18.9% in women for MBR Haemospect. The proportion of donors who would have been deferred incorrectly with haemoglobin concentration above the minimum threshold ranged from 0.1% in men for NHSBT's customary method to 20.3% in women for OrSense. Most donors preferred non‐invasive spectrometry.

**Conclusion:**

In the largest study reporting head‐to‐head comparisons of four methods to measure haemoglobin prior to blood donation, our results support replacement of NHSBT's customary method with portable haemoglobinometry.

## 1 INTRODUCTION

Blood services are mandated to measure haemoglobin concentrations of potential whole blood donors in advance of each donation. The rationale is to protect the health of donors (ie, to prevent collection from anaemic donors and mitigate the possibilities of rendering the donor anaemic) as well as to ensure the quality of blood products.^1^, ^2^ European legislation on selection criteria of blood donors (EU directive 2004/33/EC Article 4) states that haemoglobin concentration should be ≥125 g/L for women and ≥ 135 g/L for men before allowing blood donation.^3^ There is, however, substantial variation across national blood services in methods of haemoglobin measurement.^4^, ^5^ This has resulted in part because the timing of blood sampling and sample material for assessing blood donors is not defined by legislation, and partly because there is little evidence about the comparative performance of different rapid measurement methods.^6^, ^7^, ^8^, ^9^, ^10^, ^11^

The customary approach of National Health Service Blood and Transplant (NHSBT, the national blood service of England) has been a gravimetric method (copper sulphate test) carried out on finger‐prick capillary blood taken immediately before donation, followed by a spectrophotometric test (HemoCue) with venous blood for those who fail the copper sulphate test.^12^ Recent data, however, indicate that NHSBT's customary method may allow about 10% of donors to give blood despite having baseline haemoglobin concentrations below the minimum regulatory threshold.^13^, ^14^ By contrast, blood services in some countries (eg, the Netherlands and Finland) assess haemoglobin concentration before blood donation using a spectrophotometric test on capillary blood obtained by a finger‐prick.^4^ Other services (eg, France and Denmark) use haemoglobin values obtained from the most recent prior donation (“post donation” approach), employing automated haematology analysers of venous blood.^4^, ^15^ Other services (eg, Bavaria, Ireland, Spain) have employed non‐invasive spectrometry that does not require obtaining a blood sample.^4^, ^16^

We conducted a within‐person comparison of four haemoglobin measurement methods using performance metrics relevant to the blood donation context and comparing each method to the reference standard of a haematology analyser.

## 2 METHODS

### 2.1 *Study design*

**FIGURE 1:**
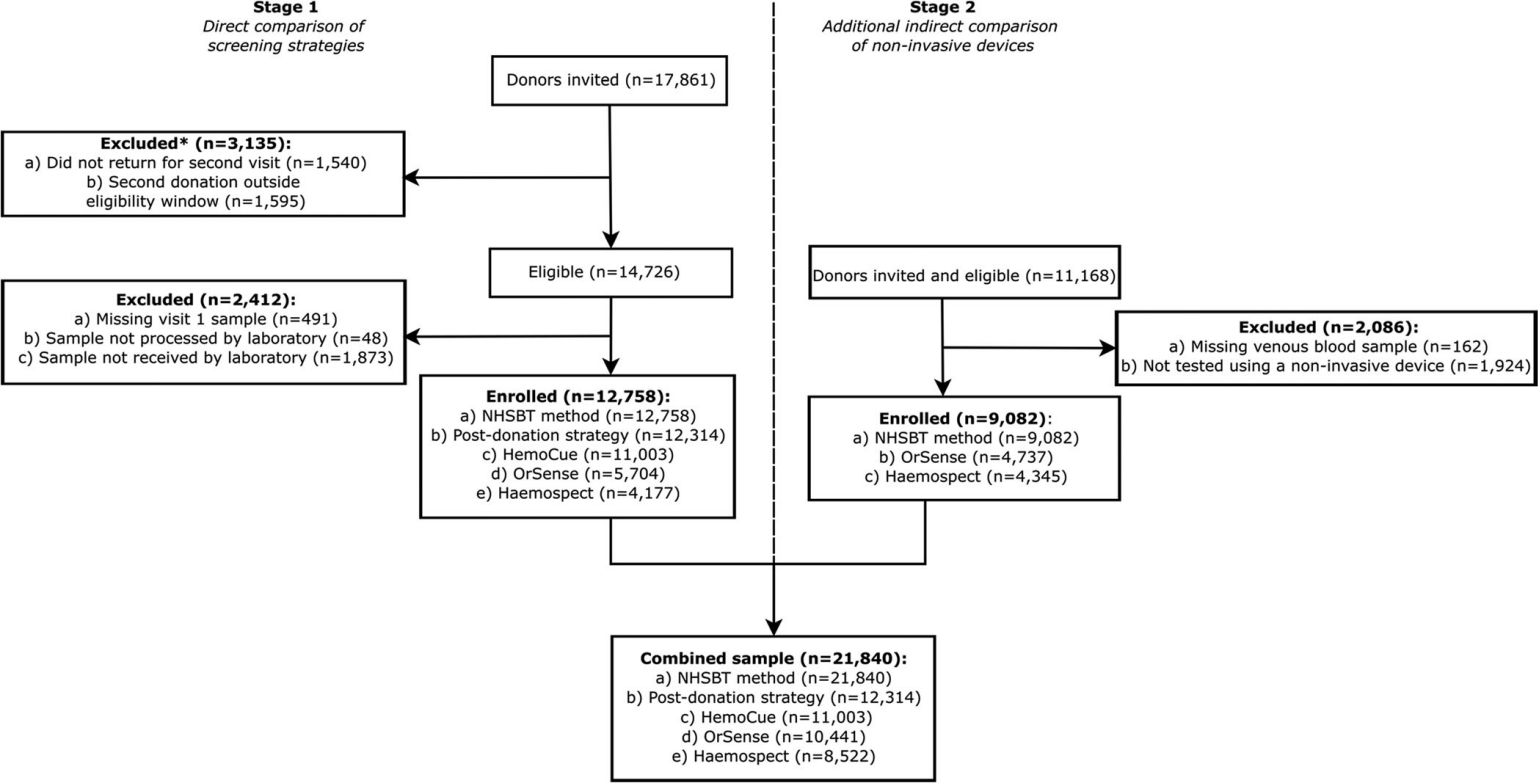
CONSORT flowchart showing. *Note*: 30% drop‐out rate expected between Stage 1 visit 1 and visit two donors as per study design

This study evaluated four haemoglobin measurement approaches used by blood services in high‐income countries (see “Diagnostic tests” below) against a haematology analyser reference standard. The study involved participant recruitment into two stages (Figure 1). Stage 1 involved direct comparisons of invasive and non‐invasive methods in the same participants. Stage 2 involved an indirect comparison of two non‐invasive spectrometry devices described below. Allocation of the non‐invasive device between teams was done by “cross‐over” randomisation. Participants in Stage 1 were not eligible to join Stage 2. The study protocol is provided in the Annex. The study was registered with ISRCTN (ISRCTN90871183), and approved by the National Research Ethics Service (15/EE/0335).

### 2.2 *Diagnostic tests*

We used haemoglobin concentration measured by a Sysmex XN‐2000 haematology analyser at a central laboratory (UK BioCentre, Milton Keynes, UK) as the study's reference standard.^17^ We evaluated four rapid diagnostic tests against that standard: (1) gravimetry/venous HemoCue (“NHSBT's customary method” at the time of this study), i.e., a copper sulphate gravimetric test carried out on finger‐prick capillary blood, followed by spectrophotometry (HemoCue AB, Ängelholm, Sweden) on venous blood for those failing gravimetry^12^; (2) “post donation” approach, that is, estimating current haemoglobin concentration from that measured by a haematology analyser at a donor's most recent prior donation (ie, about 12–16 weeks earlier); (3) “portable haemoglobinometry”, using a Hemocue 301 device using finger‐prick capillary blood^18^; and (4) one of two hand‐held non‐invasive spectrometer devices ‐ the MBR Haemospect (MBR Optical Systems GmbH & Co. KG, Wuppertal, Germany)^19^ or the Orsense NMB200 (OrSense Ltd, Petah‐Tikva, Israel).^20^

### 2.3 *Study participants*

Between February 2016 and March 2017, donors were eligible for recruitment into COMPARE if they: were aged 18 years or older; fulfilled routine criteria for donation (with the exception of pre‐donation haemoglobin concentration measured using the NHSBT testing method); had an email address and access to the internet to respond to web‐based questionnaires; and were willing to undergo additional haemoglobin concentration measurements at one of the 10 “mobile” donor centres of NHSBT, the sole blood provider to the NHS in England, UK. After reading study information leaflets and participating in a discussion with donor carer staff, eligible donors were asked to complete the study consent form and provide a blood sample. Soon after enrolment, participants received online health and lifestyle questionnaires, including the Fitzpatrick Skin Score.^21^

### 2.4 *Outcomes*

The primary endpoint was the proportion of donors in the study who would have been inappropriately bled by each method (ie, the proportion of donors for whom a given method would not identify them as having sub‐threshold haemoglobin levels as measured by the reference standard). Secondary endpoints included sensitivity, specificity, the proportion of donors who would have been excluded from blood donation (“deferred”) incorrectly, variability of the performance of different methods by donors' personal characteristics (eg, repeat vs first‐time donor, and skin colour tone), and the acceptability of different methods according to donors.

### 2.5 *Statistical analysis*

The statistical analysis followed a prespecified plan. Briefly, Bland‐Altman^22^ plots were used to assess systematic difference between haemoglobin screening methods when compared against the reference standard, and supplemented by linear regression models to examine proportional biases (ie, how much the difference between two methods is dependent on the magnitude of the measurement). The percentage of donors who would have been bled below the threshold (ie, <125 g/L for women and <135 g/L for men) was calculated by taking the number of donors categorised as having adequate haemoglobin levels by the screening method but should have been deferred according to the reference standard, and dividing by the total number of donors in the analysis population. The proportion of donors incorrectly deferred above the threshold was calculated similarly. Differences between each screening method and the reference standard were assessed using a McNemar's test for paired within‐person comparisons. For direct comparisons between strategies, donation outcomes were standardised by sex and haemoglobin level to a reference population (ie, returning Stage 1 donor population). Each observation was assigned a weight based on the relative frequency of the sex‐specific haemoglobin level appearing in the reference population relative to the estimation sample. The proportions for each of the four donation outcomes (bled below haemoglobin threshold, bled above haemoglobin threshold, deferred below haemoglobin threshold, deferred above haemoglobin threshold) were then weighted accordingly. Sensitivity (the probability of correctly identifying donors with a low haemoglobin level) and specificity (the probability of correctly identifying donors with sufficient haemoglobin levels) of each screening method were calculated and used to define the area under a receiver operating characteristic curve (AUC) to illustrate the diagnostic ability (ie, how well a test discriminates between donors with low and sufficient haemoglobin levels) of a screening method at different haemoglobin thresholds. Sex‐specific sample size was estimated to provide 80% power, at a 5% significance level, to detect a 10% relative difference in the false pass rate (ie, percentage of donors who would have been bled below the threshold) between the NHSBT customary method and any of the other tests (Annex). Analyses were conducted separately for men and women using Stata v14. The analysis adhered to the Standards for Reporting Diagnostic Accuracy Studies (STARD).^23^

### 2.6 *Role of the funding source*

The academic investigators and representatives of NHSBT, a funder of the study, participated in the study design and oversight. The investigators at the study's academic coordinating centre had sole access to the study database, and had final responsibility for data collection, data integrity, data analysis, and data interpretation, as well as manuscript drafting and the decision to submit the manuscript for publication. All authors gave approval to submit for publication.

## 3 RESULTS

A total of 29 029 participants were consented to participate in the COMPARE study (17 861 in Stage 1 and 11 168 in Stage 2), of whom 21 840 (75.2%) provided data for the current analysis (Figure 1). Table S1 shows baseline characteristics of the participants. Compared with NHSBT's general donor population, participants were, on average, older, more likely to be male, less ethnically diverse, and had a longer donation career (Tables S2 and S3). Baseline characteristics were similar between participants recruited in Stages 1 and 2, although haemoglobin concentration was approximately 3‐4 g/L lower in returning donors.

**FIGURE 2:**
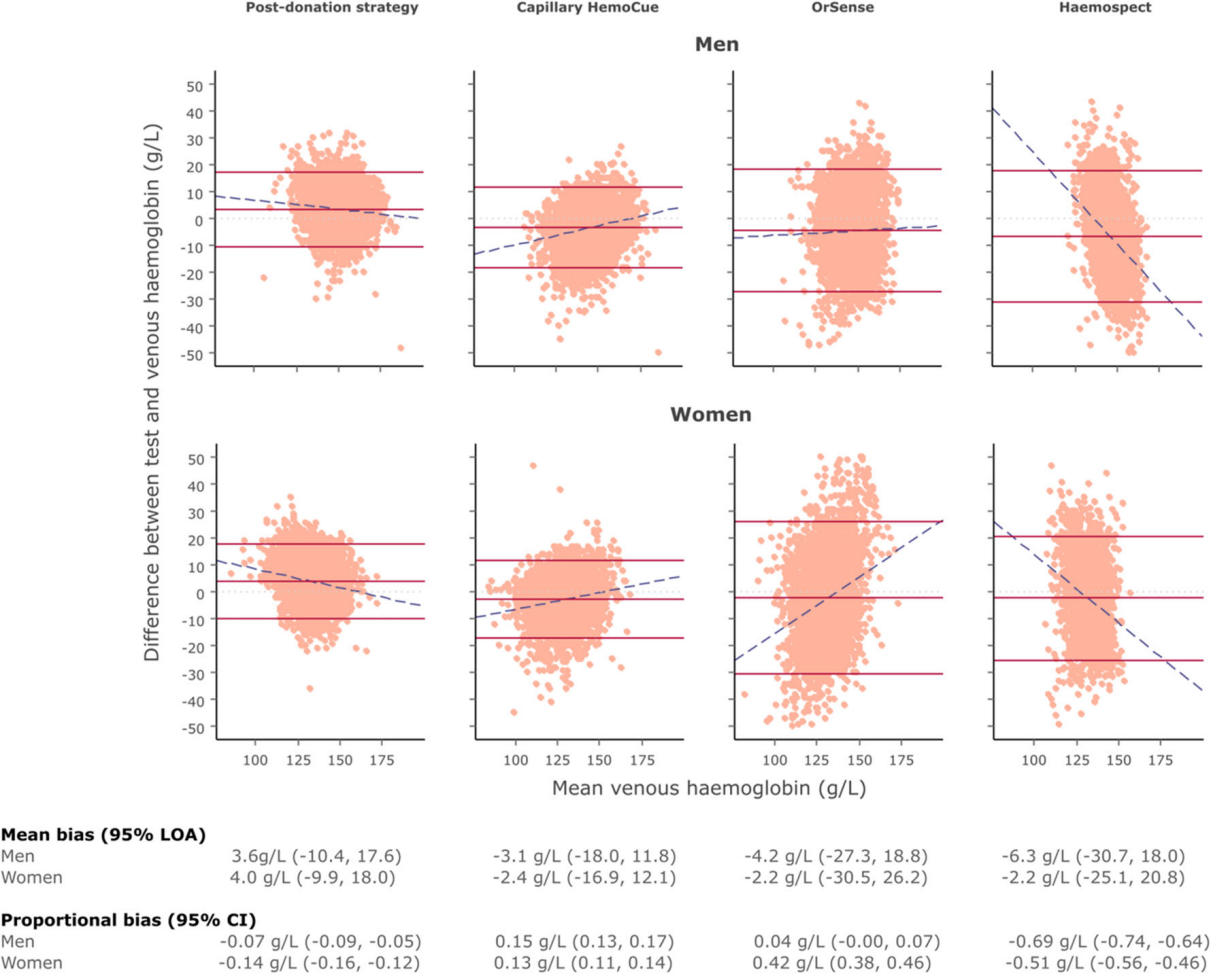
Bland–Altman plot of each haemoglobin testing strategy by sex using venous haemoglobin values as the reference test. *Note*: Dotted light grey lines represent zero bias. Solid red lines represent the mean bias of the testing strategy (middle) and accompanying 95% limit of agreement (LOA; upper and lower) of the mean bias. Dashed blue lines depict proportional bias estimated using linear regression. Men—*Post‐donation strategy*: N = 5920, 4.3% outside the LOA*. Capillary HemoCue*: N = 5279, 5.1% outside the LOA. *OrSense*: N = 4861, 5.7% outside the LOA*. Haemospect*: N = 4352, 5.5% outside the LOA. Women ‐ *Post‐donation strategy*: N = 6394, 5.2% outside the LOA. *Capillary HemoCue*: N = 5724, 5.0% outside the LOA. *OrSense*: N = 5580, 5.2% outside the LOA. *Haemospect*: N = 4170, 5.5% outside the LOA [Color figure can be viewed at wileyonlinelibrary.com]

**FIGURE 3:**
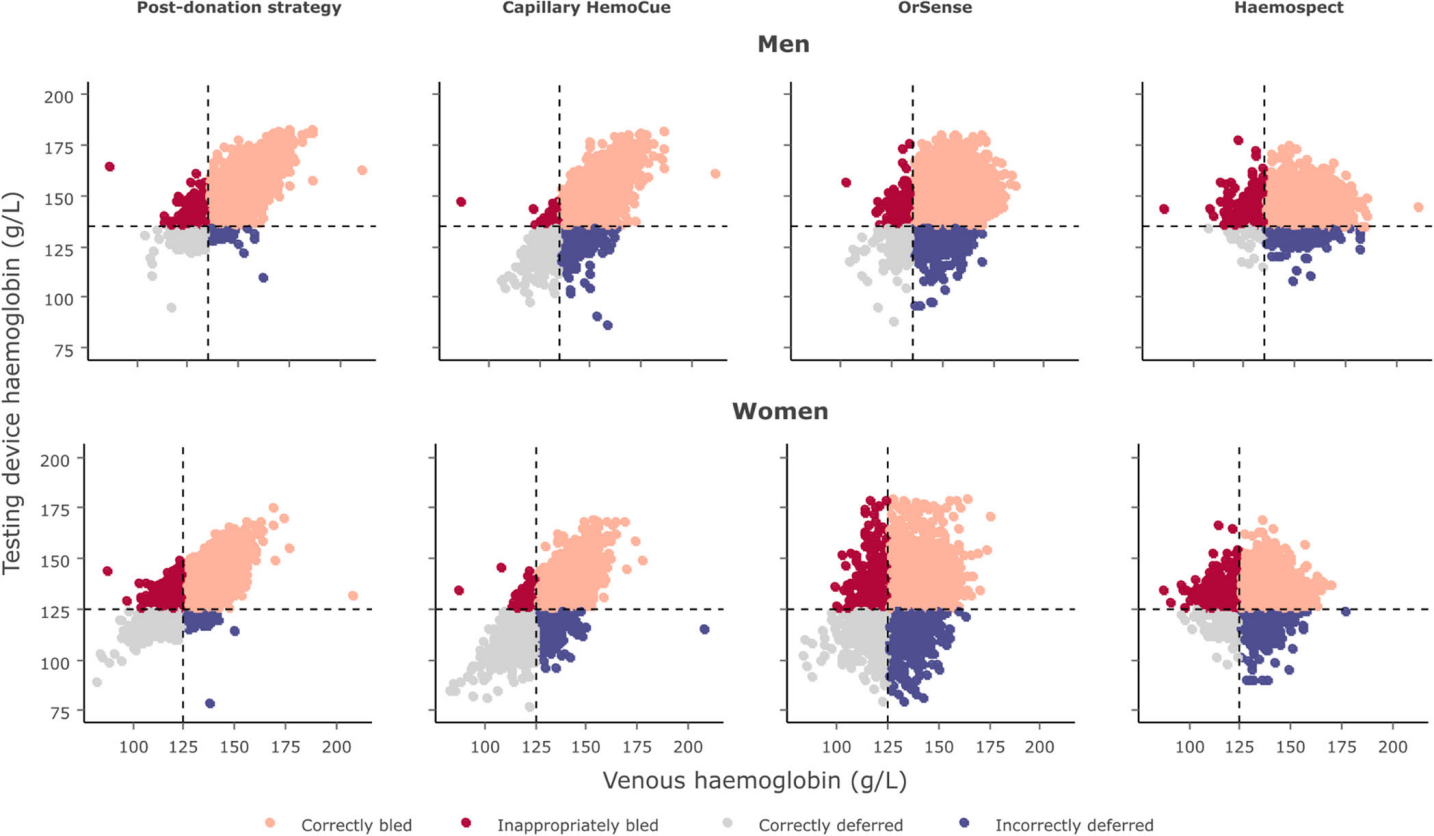
Scatterplot comparing testing device haemoglobin values to those obtained from venous blood samples by sex [Color figure can be viewed at wileyonlinelibrary.com]

Figure 2 and Figure S1 show the mean and proportional biases between the haemoglobin readings of each test and the reference standard. On average, the “post donation” approach over‐estimated haemoglobin values by 3.6 g/L (95% limit of agreement −10.4, 17.6; SD 7.1) and 4.0 g/L (−9.9, 18.0; SD 7.1) for men and women, respectively, while each of the other methods tended to under‐estimate haemoglobin values; −3.1 (−18.0, 11.8; SD 7.6) and −2.4 (−16.9, 12.1; SD 7.4) g/L for portable haemoglobinometry, −4.2 (−27.3, 18.8; SD 11.7) and −2.2 (−30.5, 26.2; SD 14.5) g/L for OrSense and −6.3 (−30.7, 18.0; SD 12.4) g/L for Haemospect. There was evidence of proportional bias for each test, with the “post donation” approach and Haemospect over‐estimating, and portable haemoglobinometry and OrSense underestimating haemoglobin levels at the lower end of the distribution. Mean biases for non‐invasive devices were larger in donors recruited in Stage 2 (Figure S2). Figures 3 and S3 show scatterplots of haemoglobin concentration measured by each testing method against the reference standard.

**FIGURE 4:**
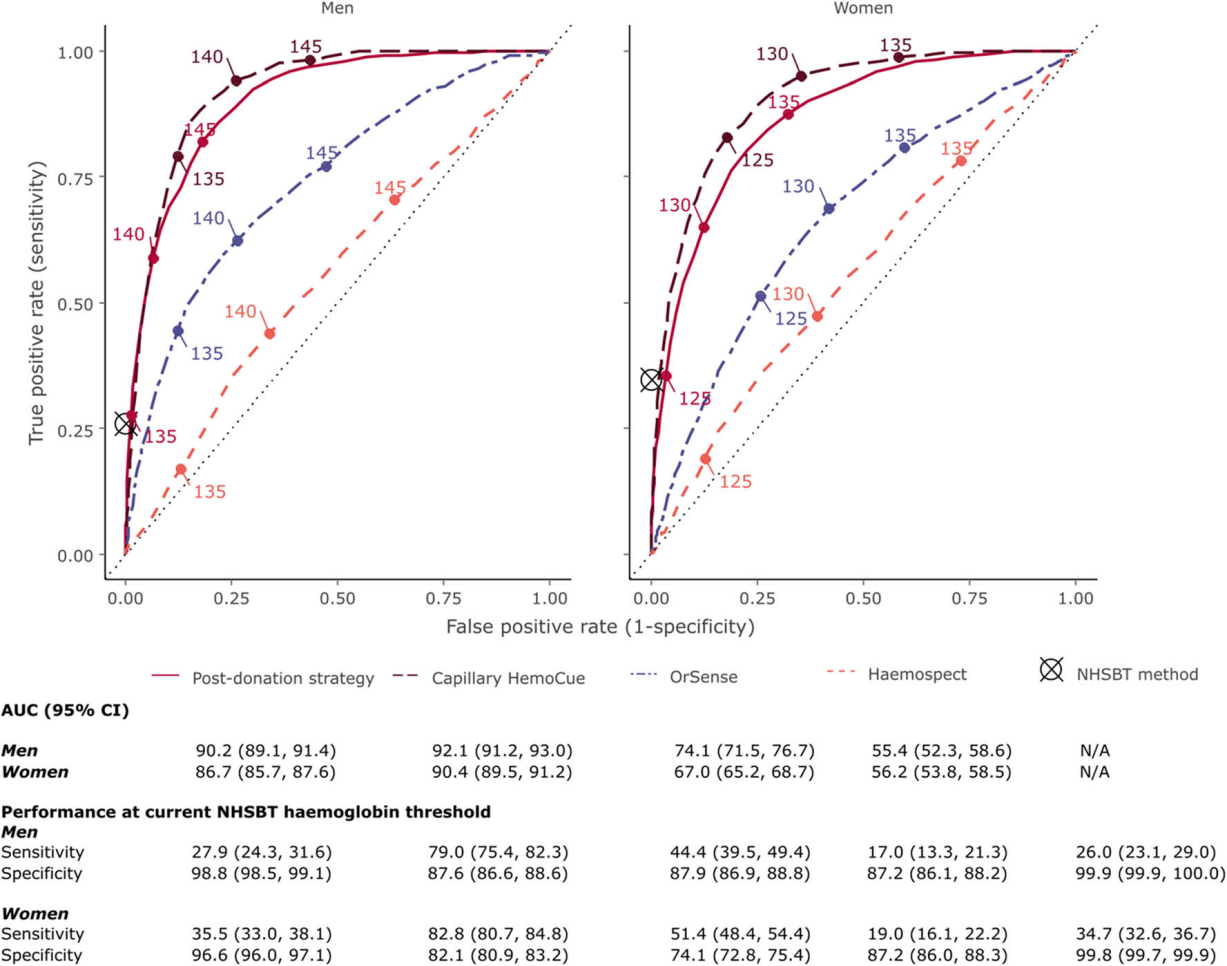
Receiver operating characteristic curves for each haemoglobin testing strategy at different haemoglobin thresholds by sex. *Note*: Threshold values are shown in g/L. Sensitivity and specificity of NHSBT method has been superimposed as it only provides a pass/fail result rather than a quantitative readout [Color figure can be viewed at wileyonlinelibrary.com]

Figure 4 shows the AUC for each test by sex. Portable haemoglobinometry had the highest AUC, for both men and women, across all haemoglobin thresholds examined, followed by the “post donation” approach, the OrSense, and Haemospect. The sensitivities of the different methods at minimum donation thresholds for men and women were 26.0% and 34.7% for NHSBT's customary method, 27.9% and 35.5% for the “post donation” approach, 79.0% and 82.8% for portable haemoglobinometry, 44.4% and 51.3% for OrSense, and 17.0% and 19.0% for Haemospect The specificity of each method at the same haemoglobin thresholds for men and women were 99.9% and 99.8%, respectively, for NHSBT's customary method, 98.8% and 96.6% for the “post donation” strategy, 87.6% and 82.1% for portable haemoglobinometry, 87.9% and 74.1% for OrSense, and 87.2% for both sexes with Haemospect (Figure S4).

**FIGURE 5:**
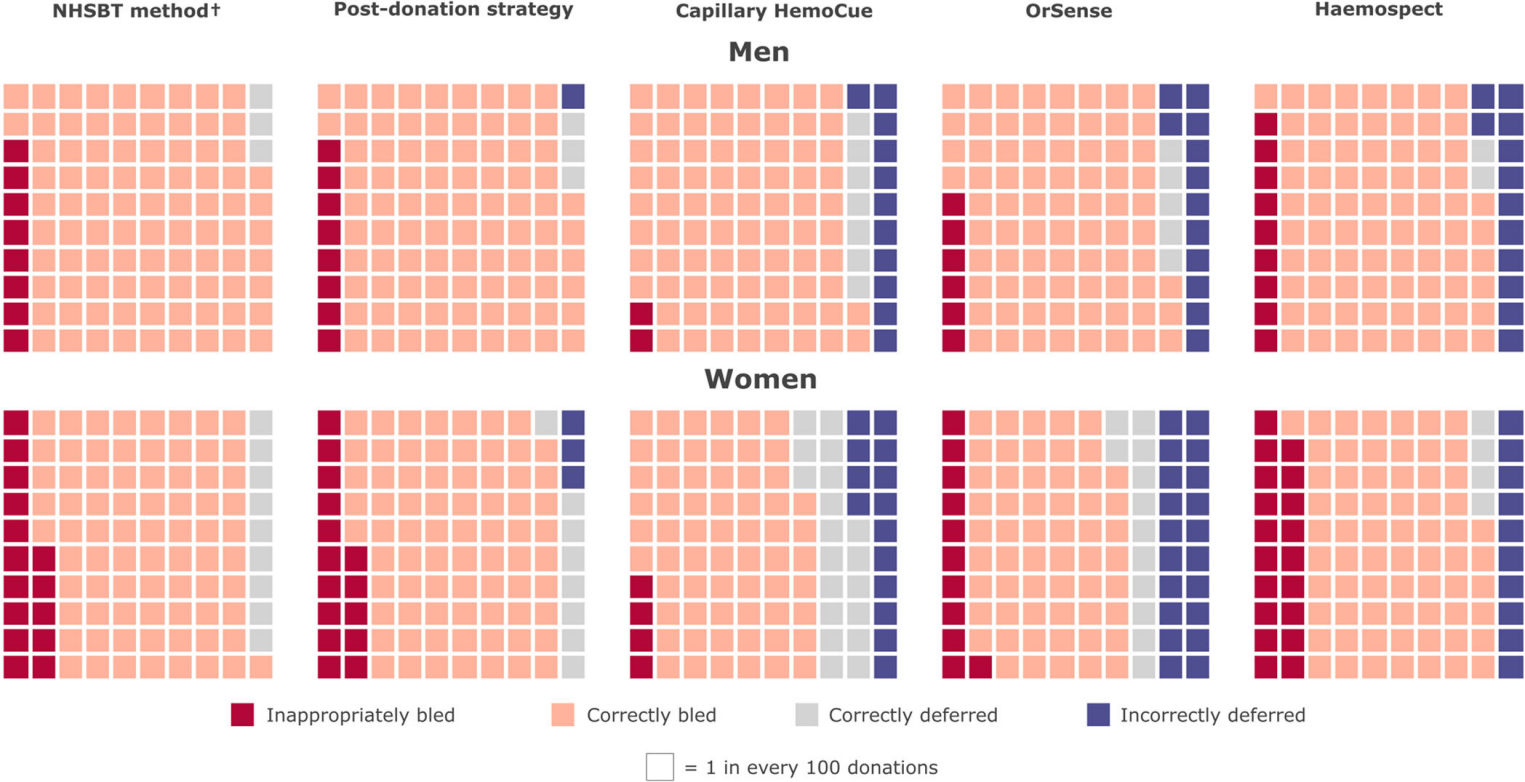
Donation outcomes by testing strategy and sex, per 100 donations standardised to the returning donor population in the COMPARE study. *Note*: ^†^1 in every 1000 donations for men, and 2 in every 1000 donations for women are incorrectly deferred using the customary NHSBT method [Color figure can be viewed at wileyonlinelibrary.com]

**FIGURE 6:**
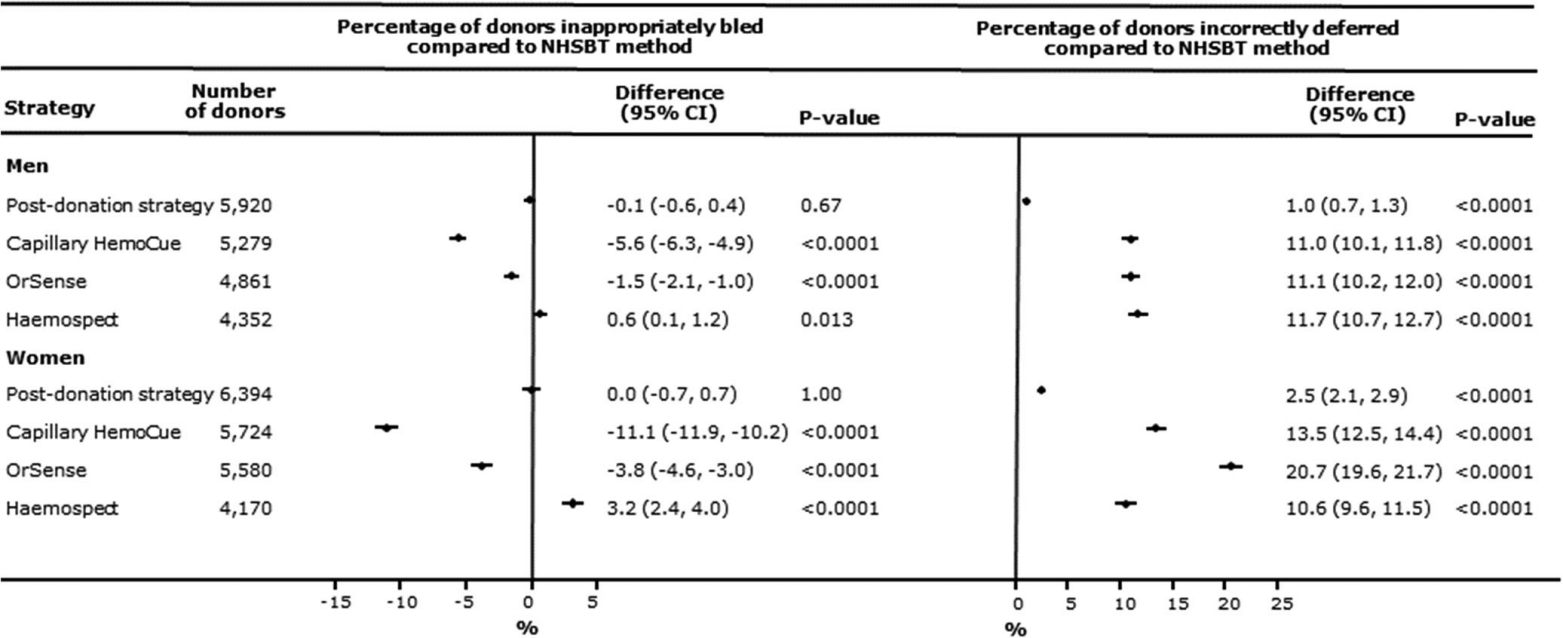
Percentage difference (95% confidence interval) in donors who would be bled below and deferred above the donation haemoglobin threshold for each testing strategy compared with the standard NHSBT test by sex. *Note: P*‐values calculated using McNemar's test

The prevalence of donors who would have been inappropriately bled ranged from 2.2% in men for portable haemoglobinometry to 18.9% in women for MBR Haemospect (Figure 5 and Table S4). Compared to NHSBT's customary method, use of portable haemoglobinometry performed best in reducing the prevalence of inappropriate bleeding (−5.6%, −6.3, −4.9 for men and −11.1%, −11.9, −10.2 for women, *P* < 0.0001 for both: Figure 6). The proportion of donors who would have been deferred with haemoglobin concentrations above the threshold ranged from 0.1% in men for NHSBT's customary method to 20.8% in women for OrSense (Figure 5 and Table S4). In a sensitivity analysis, the proportion of donors who would have been bled with haemoglobin concentrations below the minimum threshold using the “post donation” approach decreased while the number of donors inappropriately deferred somewhat increased with longer time between donation (Figure S5). There were notable differences in the accuracy of methods between white and non‐white donors, especially for the non‐invasive devices (Figure S6). Stage 1 donors lost to follow‐up tended to be on average younger, earlier in their donation career, and more likely to have had haemoglobin values beneath the threshold at their first visit (Table S6).

Regarding test acceptability, 72% of donors preferred the non‐invasive devices, 20% preferred the finger‐prick test, and 8% the “post donation” approach. However, 77% of donors reported that test accuracy was their most important consideration.

## 4 DISCUSSION

In a study of over 21 000 whole blood donors in NHSBT, the national blood service of England, we conducted head‐to‐head comparisons of four rapid methods for the measurement of pre‐donation haemoglobin levels, comparing each against the reference standard of a haematology analyser. Our key finding was that portable haemoglobinometry (ie, using the capillary HemoCue) had the highest accuracy across all haemoglobin thresholds examined for both men and women, as well as the smallest biases in comparison with the reference standard. Furthermore, pre‐specified subgroup analyses indicated that portable haemoglobinometry performed similarly well among donors of different ages, ethnicities, and levels of blood donation experience. When compared to NHSBT's customary method (ie, gravimetry/venous HemoCue), use of portable haemoglobinometry reduced the prevalence of inappropriately bled donors, but increased the proportion of donors incorrectly deferred (deferrals are disadvantageous because they demotivate donors and are costly for blood services^24^).

Based on these results, we offered two policies to NHSBT to improve its current haemoglobin screening practices. First, wholesale replacement of NHSBT's customary method with portable haemoglobinometry alone. We estimated that when projected across the approximately 1.4 million blood donations taking place annually in England, this policy would prevent about 65 000 donors annually from avoidable anaemia and potential iron deficiency and its potential consequences. A second approach would be to use portable haemoglobinometry only in donors who failed a more methodologically rigorous use of the gravimetric test. NHSBT estimated that this second approach would prevent about 30 000 donors annually from experiencing anaemia and potential iron deficiency. The second approach would avoid the higher rates of inappropriate deferrals of donors associated with the first approach. In 2018, NHSBT adopted the second approach as national policy, implementing it swiftly across the whole of the blood service of England.^25^, ^26^

We made several additional observations relevant to the policies and practices of blood services. We found that the “post donation” approach (ie, estimating current haemoglobin concentration from that measured by a haematology analyser at a donor's most recent prior donation) performed similarly to NHSBT's customary method when the interval between donations was about 12–16 weeks. However, the performance of this approach improved somewhat with longer intervals between donations, and when higher haemoglobin concentration at the first study visit was used to predict the donor's haemoglobin concentration at the next study visit. Blood services in several countries (eg, France, Denmark and Germany) have recently adopted the “post donation” approach due to its practical advantages, that is, it replaces the need for rapid on‐site testing by using a haematology analyser at a central laboratory to measure venous blood taken from the donor's sample pouch.^15^, ^27^, ^28^ Some blood services have started to supplement a post‐donation approach with monitoring of serum ferritin, a measure of the body's iron stores, in selected blood donors.^29^, ^30^, ^31^ Future work will seek to investigate the safety, cost‐effectiveness, and practicability of the “post donation” approach in large, high‐throughput blood services such as in England.

A further finding of our study was that non‐invasive spectrometry devices (ie, MBR Haemospect and Orsense NMB200) did not generally perform well compared with the other methods, despite their obvious advantage of avoiding the need to take a blood sample. For example, these methods showed lower sensitivity for detection of haemoglobin concentration below the threshold for donation than portable haemoglobinometry, meaning higher numbers of donors would be inappropriately bled. Furthermore, non‐invasive spectrometry devices, which measure haemoglobin by shining light on the skin of donors, performed inconsistently in people of different ethnicities and skin colour types, limiting the test's potential applicability to blood services in countries with a large and ethnically diverse pool of donors such as in the UK. Some blood services have suffered adverse consequences from introducing non‐invasive spectrometry without such robust assessment.^16^ Our study showed estimates of haemoglobin concentration by non‐invasive methods, which would result in higher levels of inappropriate bleeding and/or higher levels of inappropriate deferral in blood donors when compared with portable haemoglobinometry. Nevertheless, further efforts are warranted to improve the performance of non‐invasive spectrometry devices, given their potential to enhance the experience of blood donation by avoiding pain.

The current study had major strengths. It involved large numbers of participants, providing excellent statistical power and detailed comparisons of important sub‐populations (eg, sex‐specific results). The study design was a within‐person comparison, enhancing validity by providing head‐to‐head comparisons of different methods to measure haemoglobin concentrations. It involved evaluation of four methods, making it wider in scope than previous efforts focusing on fewer methods.^7^, ^9^, ^32^, ^33^, ^34^ It used a state‐of‐the‐art haematology analyser in an accredited central laboratory as the reference standard. The study was embedded in NHSBT's routine blood service, enabling rapid recruitment of blood donors and resulting in findings of direct relevance to UK blood services.

Our study also had potential limitations. First, only about three‐quarters of participants initially consented into the study returned for the second visit to allow measurements of haemoglobin concentration for the study purpose; however, a non‐attendance rate of 30% at the second visit was originally factored into power calculations. Second, compared to the national donor population in England, participants in the study were older, more likely to be male, less ethnically diverse, and had a longer blood donation career. Hence, some caution is needed in extrapolating the findings to the general population of blood donors. Third, when assessing the post‐donation approach we invited participants for a second visit about 12–16 weeks later, meaning our study had limited ability to assess this method for longer inter‐donation intervals. Fourth, the study recruited only a limited number of non‐white participants and relied on self‐reported information for skin colour tone, limiting ability to assess potential differences by ethnic background.

In summary, in the largest study reporting head‐to‐head comparisons of four methods to measure haemoglobin prior to blood donation, our results support replacement of NHSBT's customary method with portable haemoglobinometry.

## CONFLICT OF INTEREST

John Danesh reports grants, personal fees and non‐financial support from Merck Sharp & Dohme (MSD), grants, personal fees and non‐financial support from Novartis, grants from Pfizer and grants from AstraZeneca outside the submitted work.

John Danesh serves on the International Cardiovascular and Metabolic Advisory Board for Novartis (since 2010); the Steering Committee of UK Biobank (since 2011); the MRC International Advisory Group (ING) member, London (since 2013); the MRC High Throughput Science Omics Panel Member, London (since 2013); the Scientific Advisory Committee for Sanofi (since 2013); the International Cardiovascular and Metabolism Research and Development Portfolio Committee for Novartis; and the AstraZeneca Genomics Advisory Board (2018).

## AUTHOR CONTRIBUTION

All authors contributed to data collection, study design, data analysis, interpretation, and drafting of this paper.

David J Roberts, John Danesh, Emanuele Di Angelantonioon are joint last authors.

Steven Bell and Michael Sweeting are joint first authors.

Writing Committee: Steven Bell, Michael Sweeting, Anna Ramond, Ryan Chung, Stephen Kaptoge, Matthew Walker, Thomas Bolton, Jennifer Sambrook, Carmel Moore, Amy McMahon, Sarah Fahle, Donna Cullen, Susan Mehenny, Angela M Wood, Jane Armitage, Willem H Ouwehand, Gail Miflin, David J Roberts, John Danesh, Emanuele Di Angelantonio Study Steering Committee: Jane Armitage FRCP (independent chair), University of Oxford; John Danesh FMedSci, University of Cambridge; Emanuele Di Angelantonio FRCP, University of Cambridge and NHSBT; Jenny Donovan FMedSci (independent member), University of Bristol; Ian Ford FRSE (independent member), University of Glasgow; Rachel Henry, University of Cambridge; Beverley J Hunt FRCPath (independent member), King's College, London; Bridget le Huray (lay member); Susan Mehenny, NHSBT; Gail Miflin FRCPath, NHSBT; Amy McMahon, PhD, University of Cambridge; Carmel Moore PhD, University of Cambridge; Willem H Ouwehand FMedSci, University of Cambridge and NHSBT; Jane Green, NHSBT; David J Roberts FRCPath, University of Oxford and NHSBT; Mike Stredder, NHSBT; Simon G Thompson FMedSci, University of Cambridge; Matthew Walker PhD, University of Cambridge; Nicholas A Watkins PhD, NHSBT. Previous members: Alan McDermott, NHSBT; Clive Ronaldson, NHSBT; Claire Thomson MSc, University of Cambridge; Zoe Tolkien PhD, University of Cambridge; Lorna Williamson FRCP, NHSBT; Simon G Thompson FMedSci, University of Cambridge; Jane Green, NHSBT; Study Management Committee: Lindsay Bissell, NHSBT; Donna Cullen, NHSBT; Gavin Cho MD, NHSBT; Emanuele Di Angelantonio FRCP, University of Cambridge and NHSBT; Susan Mehenny, NHSBT; Amy McMahon, PhD, University of Cambridge; Carmel Moore PhD, University of Cambridge; Hannah Perry, MSc, NHSBT; Jennifer Sambrook PhD, University of Cambridge; Michael Sweeting, PhD, University of Cambridge; Matthew Walker PhD, University of Cambridge. Haematology Review Group: David Allen DPhil, University of Oxford and NHSBT; David Bruce FRCPath, Oxford University Hospitals NHS Foundation Trust; Fizzah Choudry MRCP, University of Cambridge; Emanuele Di Angelantonio FRCP, University of Cambridge and NHSBT; Cedric Ghevaert FRCPath, University of Cambridge; Kirstie Johnston, NHSBT; Anne Kelly PhD, University of Cambridge; Andrew King FRCPath, Weatherall Institute of Molecular Medicine, University of Oxford; Susan Mehenny, NHSBT; Gail Miflin FRCPath, NHSBT; Alfred Mo MRCGP, NHSBT; Carmel Moore PhD, University of Cambridge; Willem H Ouwehand (co‐chair) FMedSci, University of Cambridge and NHSBT; Lizanne Page LMSSA, NHSBT; Penny Richardson, NHSBT; David J Roberts (co‐chair) FRCPath, University of Oxford and NHSBT; Jennifer Sambrook PhD, University of Cambridge; Peter Senior, NHSBT; Yagnesh Umrania PhD, University of Cambridge; Matthew Walker PhD, University of Cambridge; Henna Wong FRCPath, Oxford University Hospitals NHS Foundation Trust. NHSBT Mobile Donation Teams (managers): Coventry (Adele Lapworth); Herts (Emma Martin/Victoria Riggs); Lancaster (Carolyn Lancaster/Nicola Holland); Leeds (Elizabeth Copp); Norwich (Jackie Barber); Oxford (Charlotte Blake); Sheffield North (Alan Ball/Jennifer Mullins); South Anglia (Jenny Jones); Sutton Coldfield (Jane Whistance); Teesside (Clare McNally). NHSBT COMPARE Study Administration Team: Joanne Addy, Patricia Barrass, Louise Stennett. COMPARE Helpdesk: Susan Burton, Hannah Dingwall, Rachel Henry. Data Management Team: Matthew Walker, Thomas Bolton, Michael Daynes UK Biocentre: Samples were processed at the National Biosample Centre (www.ukbiocentre.com). Mark Beggs, Bradley Dowle, Phil Eeles, James Fenton, Suresh George, Chrysanthi Gilantzi, Zoe Hewitson, Ian Jarvis, Lisa Kirkwood, Lori Lewis, Katherine McBirney, Andrew Nicholson, Kristian Spreckley. Study Statisticians: Stephen Kaptoge PhD, University of Cambridge; Ryan Chung, University of Cambridge; Michael Sweeting PhD, University of Cambridge.

Co‐investigators: Michael Sweeting, Anna Ramond, Stephen Kaptoge, Matthew Walker, Jennifer Sambrook, Carmel Moore, Amy McMahon, Sarah Fahle, Donna Cullen, Susan Mehenny, Angela M Wood, Willem H Ouwehand, Gail Miflin, David J Roberts. Chief Investigators: Emanuele Di Angelantonio; John Danesh.

## Supporting information


**Appendix S1:** Supporting information
